# Supplementary figures and images for: PhysioFit: a software to quantify cell growth parameters and extracellular fluxes
Source: Bioinformatics. 2024 Jul 29;40(8):btae488. doi: 10.1093/bioinformatics/btae488 (PMC11303505; doi:10.1093/bioinformatics/btae488)

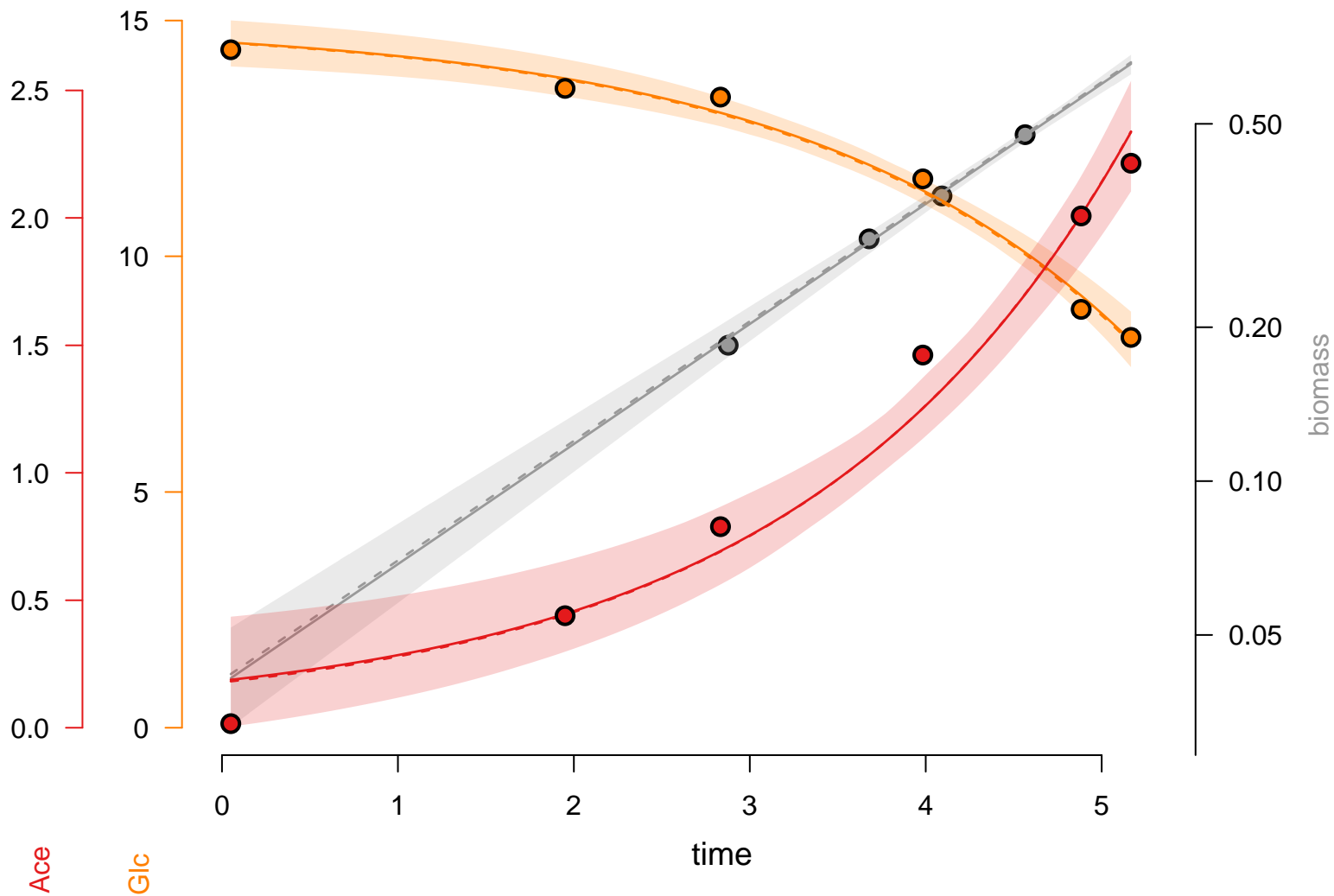

Glc, Ace concentration

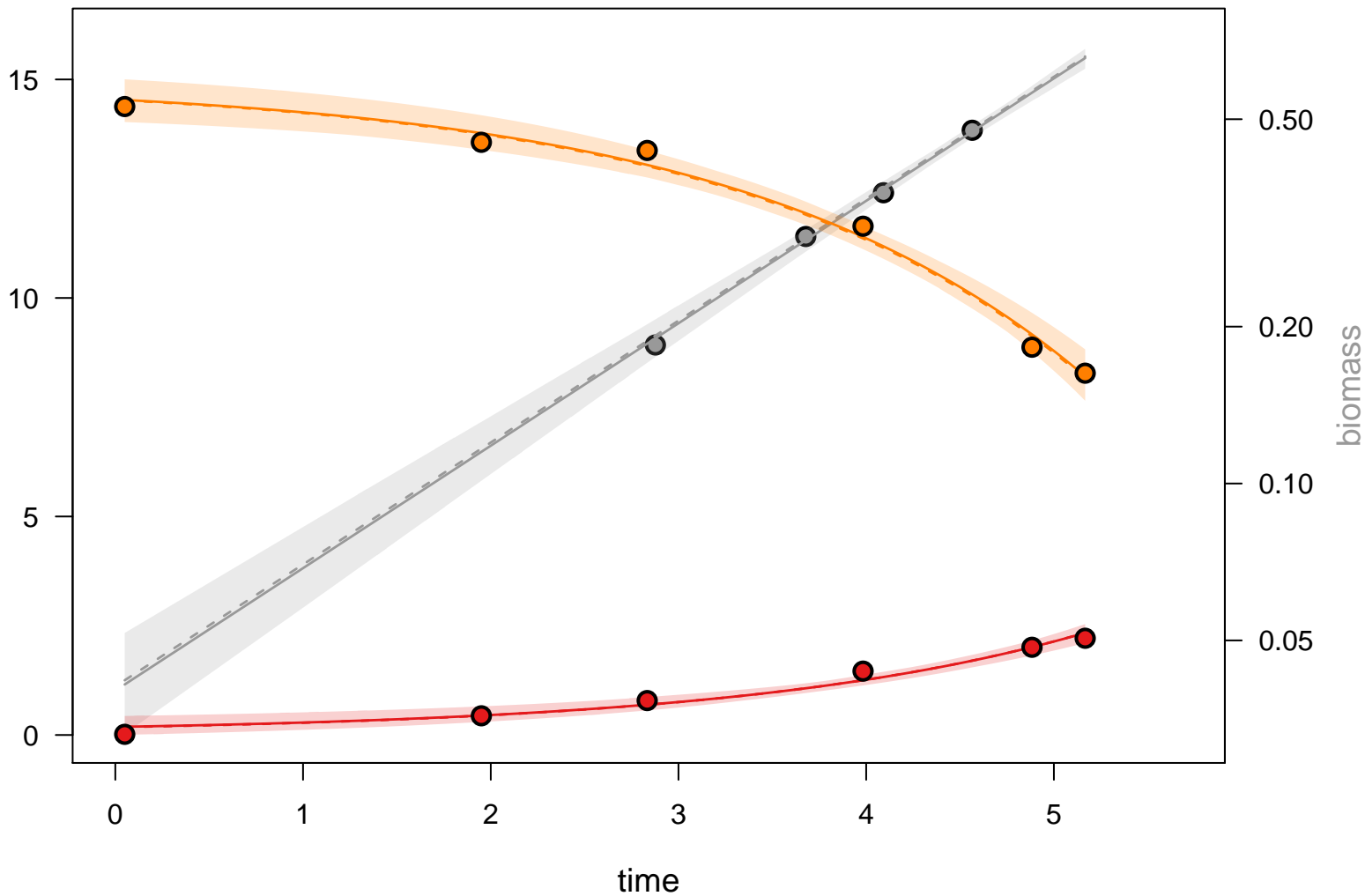

measured – simulated

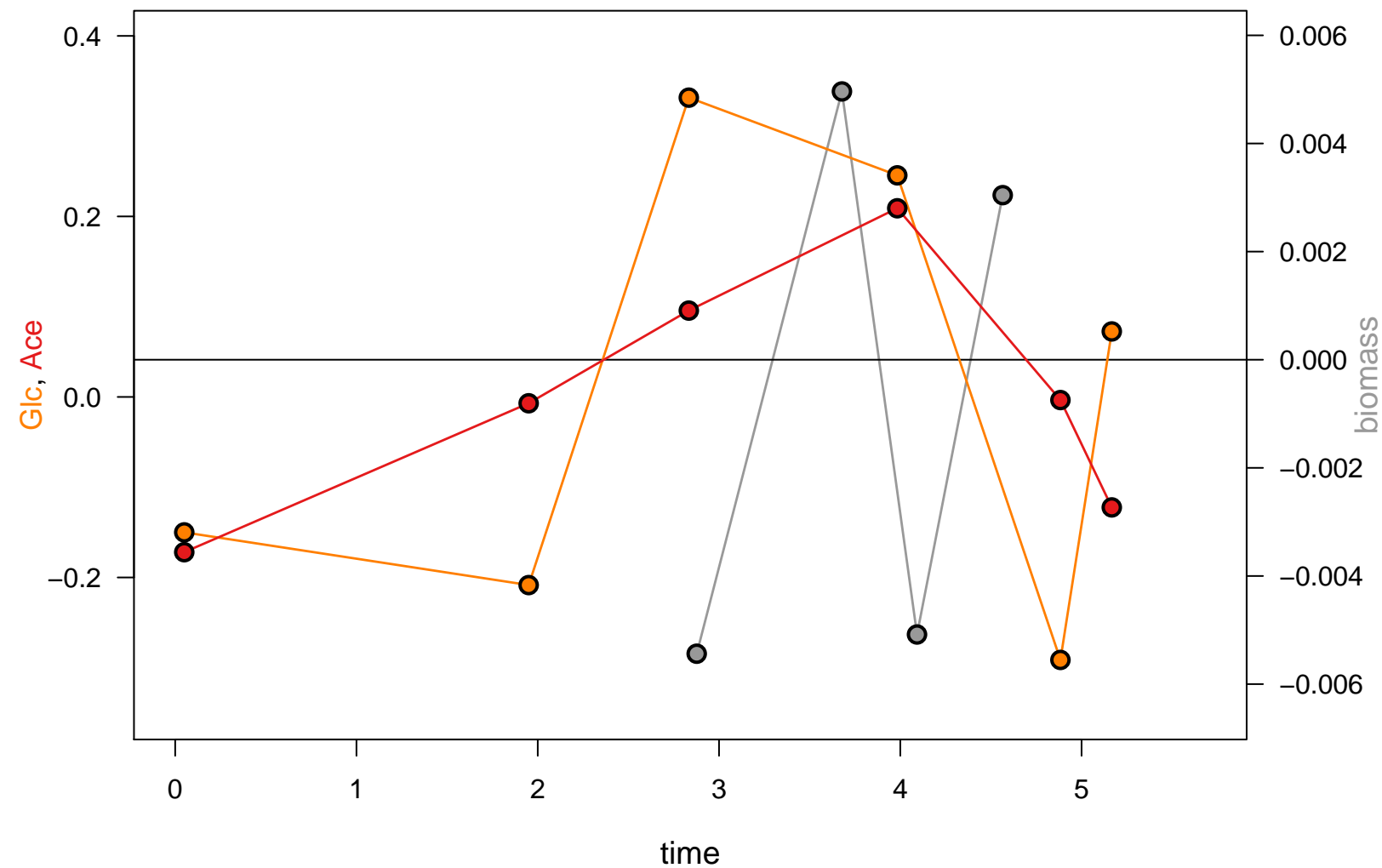

Supplement: btae488_Supplementary_Data [file btae488_supplementary_data.zip › Supplementary_information/validation_results/Berges_2021/Berges_2021/artI_res/8.pdf]
